# Acute Myeloid Leukemia With Systemic Lupus Erythematosus: Rare Association or Coincidence

**DOI:** 10.7759/cureus.20714

**Published:** 2021-12-26

**Authors:** Samah Tahri, Habiba Alaoui, Houda Bachir, Siham Hamaz, Khalid Serraj

**Affiliations:** 1 Internal Medicine, Faculty of Medicine and Pharmacy, Mohammed First University, Oujda, MAR; 2 Internal Medicine, Medical School of Oujda, Oujda, MAR; 3 Internal Medicine, Medical School of Oujda, Mohammed First University, Oujda, MAR

**Keywords:** lymphopenia, pancytopenia, association, acute myeloid leukemia, systemic lupus erythematosus

## Abstract

Systemic lupus erythematosus (SLE) is a chronic autoimmune disorder due to autoantibodies directed against nuclear and cytoplasmic antigens that may affect several different organs. The association of SLE and acute myeloid leukemia (AML) is rare, the incidence of this combination is not known, but there are few case reports in the literature. We report here the case of a 62-year-old woman, admitted for aetiological diagnosis of weight loss and severe anemic syndrome. The blood count has objectified a pancytopenia with lymphopenia. A thorough assessment was carried out following which a diagnosis of AML associated to SLE was retained. The patient received a low dose of cytarabine due to comorbidities and poor performance status associated with steroids and she died three months after diagnosis with a septic shock.

## Introduction

There are few studies and case reports about the association of AML-SLE (acute myeloid leukemia-systemic lupus erythematosus), mostly as part of the follow-up of patients already diagnosed for SLE. The increase of the risk of hematologic malignancies including AML in patients with SLE was demonstrated in few meta-analyses, but the incidence of association of AML-SLE remains unknown. The first case of this rare condition was published by Lee in 1955 [1], the last one reported was in 2018 by Massi et al. [2]. The immunosuppressive treatment of SLE is often incriminated in the development of hematologic malignancies in patients previously diagnosed and treated for SLE. This is not the case of our patient in whom the diagnosis of both diseases was simultaneous. We report here a case of rare association of AML-SLE diagnosed simultaneously.

## Case presentation

We present the case of a 62-year-old woman, known hypertensive for 10 years with ischemic heart disease under treatment by acetylsalicylic acid, carvedilol and amlodipine. This patient is also a known epileptic for 15 years under valproic acid and phenobarbital, who presented one month before her admission with a history of weight loss estimated at seven kilograms (7 kg), a severe anemic syndrome with important asthenia, skin pallor, headache and palpitations along with intermittent fever.

**Figure 1:**
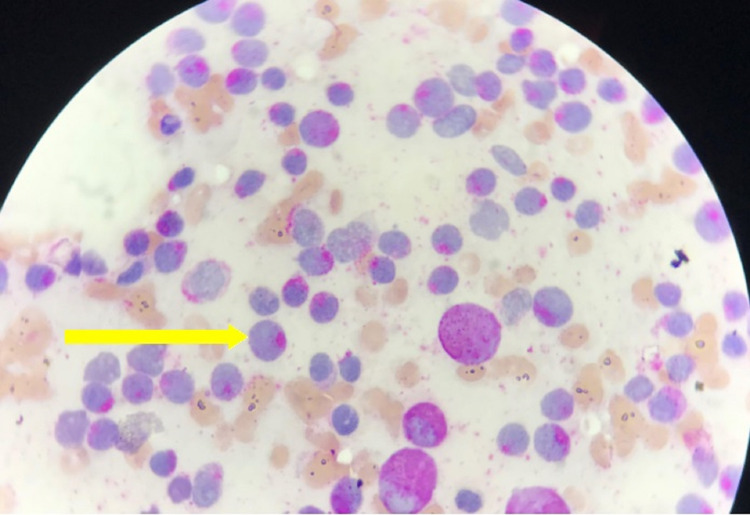
MPO positive reaction (yellow arrow) MPO: Myeloperoxydase

**Figure 2:**
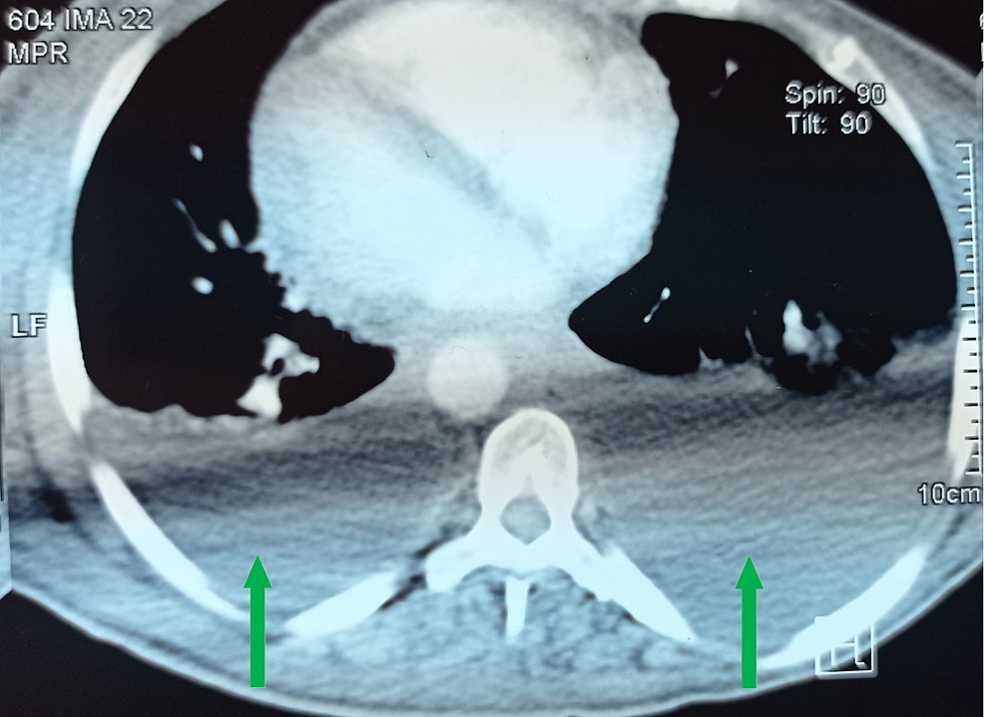
Cross-sectional thoracic CT scan showing bilateral pleural effusion (green arrows). CT: Computed Tomography

**Figure 3:**
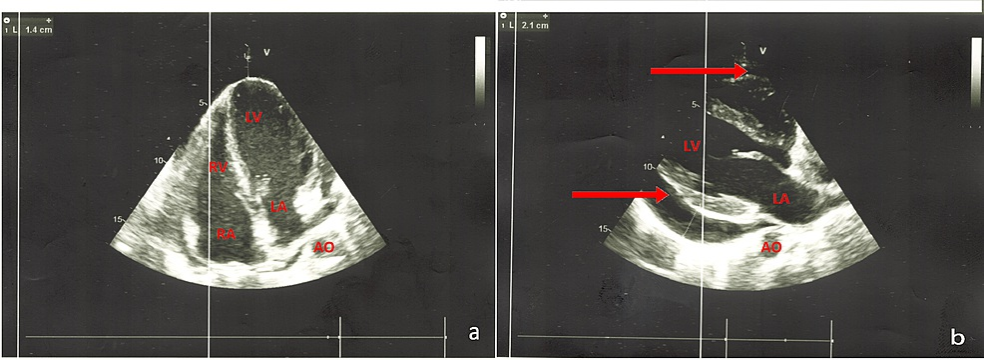
a: Repercussion on the right cavities. b: Circumferential pericardial effusion (red arrows). Left ventricle filling pressure is normal. Good left ventricle systolic-diastolic function. LV: left ventricle, LA: left atrium, RV: right ventricle, RA: right atrium, AO: aorta

Physical examination showed a conscious patient 15 out of 15 on Glasgow Coma Scale (GCS) with a Performance status score at 2, severe pallor, discolored conjunctiva, edema of lower limbs reaching knees, febrile at 39.4° Celsius, blood pressure at 100/60mmhg (millimeter of mercury), respiratory rate at 21 cycles per minute, and pulse rate at 120 beats per minute. Heart and lung auscultation was normal. No evidence of skin or mucosal bleeding. No lymphadenopathy or hepatosplenomegaly was found. Peripheral pulses were present and symmetrical. The blood count has shown anemia with 7g/dl of hemoglobin, the mean corpuscular volume (MCV) at 86 fl, reticulocyte rate at 44000/mm^3^, thrombocytopenia with 30000/mm^3^ of platelets, leukopenia with neutrophils at 40/mm^3^ and lymphocytes at 520/mm^3^. The blood smear objectified blastoid cells. The bone marrow aspiration showed 65% of myeloid blast cells. The immunophenotyping confirmed the acute myeloid leukemia diagnosis: MPO (myeloperoxidase) (Figure 1), CD (cluster of differentiation) 13, CD33, CD117 and CD34 were positive. Medullary karyotype was refused by the patient. The sedimentation rate was raised to 90mm/hour. C-reactive protein was elevated to 188 mg/l. Concerning the etiological assessment of edema, a 24-hour proteinuria assay was estimated and found to be increased at 1.4g/24 hours and albumin serum was low at 20g/l. The thoraco-abdominal-pelvic scan showed moderate bilateral pleural effusion (Figure 2) and pericardial effusion. Transthoracic echocardiography confirmed the pericardial effusion (Figure 3) and showed bilateral atrial dilatation with conserved ejection fraction at 55%. An anti-nuclear antibodies assay was positive at 1/1280 (by indirect immunofluorescence) with speckled appearance. The antibodies typing revealed positive anti-SSA (Sjogren’s syndrome type A antigen), anti-SSB (Sjogren’s syndrome type B antigen) and anti-DNA (Deoxyribonucleic Acid antibodies). Anti-phospholipid antibodies were positive for circulating lupus-type antibodies. C3-C4 complements were not consumed. Direct Coombs test was positive. Viral hepatitis (B and C viruses) and HIV serology tests were negative. The search of tuberculosis was negative. The patient's results and references values are resumed in Table 1.

**Table 1:** Laboratory test results of the patient g: gram, mg: milligram, pg: picogram, L: liter, dL: deciliter, µL: microliter, fL: femtoliter, µm3: cubic micrometer, mm3: cubic millimeter, mmol: millimole, µmol: micromole, nmol: nanomole, mm: millimeter, G: Giga.

	Patient results	Reference values
Hemoglobin	7g/dL ─ 4.3mmol/L	12-16g/dL ─ 7.5-9.9mmol/L
MCV (Mean Corpuscular Volume)	86fL ─ 86µm^3^	80-98fL ─ 80-98µm^3^
MCH (Mean Corpuscular Hemoglobin)	28pg/cell	27-32pg/cell
Reticulocyte rate	44000/µL ─ 44G/L	20000-80000/µL ─ 20-80G/L
Platelets	30000/µL ─ 30G/L	150000-400000/µL ─ 150-400G/L
Neutrophils	40/µL ─ 40/mm^3^	1500-7000/µL ─ 1500-7000/mm^3^
Lymphocytes	520/µL ─ 520/mm^3^	1000-4000/µL ─ 1000-4000/mm^3^
C-reactive protein	188mg/L ─ 1790nmol/L	0-5mg/L ─ 0-47nmol/L
Erythrocyte Sedimentation rate at first hour	90mm	F <30mm M<20mm
Creatinine	5mg/L ─ 44µmol/L	5.7-11.1mg/L ─ 50-98µmol/L
24 hours proteinuria	1400mg/24hours	10-150mg/24hours
Serum Albumin	20g/L ─ 2g/dL	35-50g/L ─ 3.5-5.5g/dL
C3 complement	1.33g/L	0.82-1.93g/L
C4 complement	0.31g/L	0.15-0.57g/L

According to the 2019 EULAR (The European Alliance of Associations for Rheumatology)/ACR (American College of Rheumatology) classification we can retain the SLE with renal involvement. This diagnosis was concomitant to AML diagnosis.

In terms of therapy, the patient received a low dose of cytarabine for AML considering the general state with Karnofsky score at 30% and the comorbidities. For the SLE, the renal biopsy could not be done due to the deep thrombocytopenia. Corticosteroid (prednisone) at One milligram per kilogram per day (1mg/kg/day) was given without any improvement.

Ten days after the end of the first chemotherapy cure, the patient presented a fever with elevation of C-reactive protein to 300mg/l. Urine culture was sterile. Chest radiography was normal. Blood culture was positive to *Klebsiella spp* and the patient was put on antibiotics adapted to the antibiogram. Despite the antibiotic treatment, the patient has died with a septic shock shortly after.

## Discussion

The studies of the association of AML-SLE are rare, but this association is increasing with time. A case-control study based on Swedish registers was published in 2009 by Lofstrom et al. to assess the risk factors for leukemic transformation and myeloid leukemia in patients with SLE. This study includes a cohort of 6438 patients over a period of 31 years. Only six patients were diagnosed with AML (0.09%) [3]. Another study published in 2013 by Lu et al. suggested that only seven patients had AML from a large SLE cohort of 16409 patients (0.04%) [4].

The physiopathology of this association is still unclear but there are multiple hypotheses. Some studies suggested the existence of hematological aberrations and or myelodysplastic syndrome associated with a possible transition to AML hence the interest of bone marrow study in patients with SLE especially in case of persistent leucopenia or anemia [3]. Other studies incriminate SLE immunosuppressive treatments such as the alkylator agent cyclophosphamide and the anti-metabolite azathioprine as a risk factor for Leukemia in SLE patients [5,6]. On the other hand, anti-malarial drugs like chloroquine, often used in patients with SLE, have been reported to exert protective actions by preventing mutations and improving cellular mechanisms of DNA (deoxyribonucleic acid) repair that can explain in part the rare association [7]. Our patient was diagnosed with both AML and SLE at the same time which makes the first hypothesis more probable in this case.

The therapeutic management of AML in SLE is very difficult in absence of a consensus because of the comorbidities (kidney and lung involvement, immune deficiency…). The main complication in patients with AML undergoing chemotherapy is febrile neutropenia that is majored by the immune deficiency in patients with AML-SLE. In our case, the patient has received palliative treatment with low-dose cytarabine and she presented a septic shock.

The prognosis of the association of AML-SLE is poor, the median survival time after the diagnosis of AML in SLE patients is seven months (<2-18 months) [3]. Our patient has died three months after the diagnosis by a septic shock.

## Conclusions

The association of AML-SLE is very rare and simultaneous diagnosis is probably a coincidence. The diagnosis is difficult because of the common hematological abnormalities seen in both diseases. Immunosuppressive treatment is the main risk factor. The prognosis of this association is very poor. Therapeutic management is a big issue because of the multiple comorbidities. The main complication and cause of death is infection.
